# InAs on GaAs Photodetectors Using Thin InAlAs Graded Buffers and Their Application to Exceeding Short-Wave Infrared Imaging at 300 K

**DOI:** 10.1038/s41598-019-49300-z

**Published:** 2019-09-06

**Authors:** Soo Seok Kang, Dae-Myeong Geum, Kisung Kwak, Ji-Hoon Kang, Cheol-Hwee Shim, HyeYoung Hyun, Sang Hyeon Kim, Won Jun Choi, Suk-Ho Choi, Min-Chul Park, Jin Dong Song

**Affiliations:** 1 Center for Opto-Electronic materials and devices, Korea Institute of Science and Technology, Seoul, 136-791 Republic of Korea; 2 Department of Applied Physics and Institute of Natural Sciences, Kyung Hee University, Yongin, 17104 Republic of Korea; 3 Advanced Analysis Center, Korea Institute of Science and Technology, Seoul, 136-791 Republic of Korea

**Keywords:** Composites, Sensors and biosensors

## Abstract

Short-wave infrared (SWIR) detectors and emitters have a high potential value in several fields of applications, including the internet of things (IoT) and advanced driver assistance systems (ADAS), gas sensing. Indium Gallium Arsenide (InGaAs) photodetectors are widely used in the SWIR region of 1–3 μm; however, they only capture a part of the region due to a cut-off wavelength of 1.7 μm. This study presents an InAs p-i-n photodetector grown on a GaAs substrate (001) by inserting 730-nm thick In_x_Al_1−x_As graded and AlAs buffer layers between the InAs layer and the GaAs substrate. At room temperature, the fabricated InAs photodetector operated in an infrared range of approximately 1.5–4 μm and its detectivity (D^*^) was 1.65 × 10^8^ cm · Hz^1/2^ · W^−1^ at 3.3 μm. To demonstrate performance, the Sherlock Holmes mapping images were obtained using the photodetector at room temperature.

## Introduction

Indium Gallium Arsenide (InGaAs) photodetectors on Indium Phosphide (InP) substrates are commonly used to detect the ‘classic’ short-wave infrared (SWIR) range of 1–1.7 μm. Such devices are widely used in spectroscopy, night vision, gas sensing and telecommunications applications^1–5^.

Recently, a ‘capacity crunch’ in this wavelength range was predicted based on the explosive increase in network traffic that is being driven by the development of the internet of things (IoT). Systems such as fiber optics, waveguides, emitters, and detectors are undergoing extensive research to find ways to extend performance from 1.7 μm to longer wavelengths in the SWIR range^6–11^. Such extensions could support a variety of applications, including pedestrian detection for advanced driver assistance systems (ADAS), and the identification of particulate matter in the air^12^.

HgCdTe (MCT) detectors are currently in widespread use in the SWIR range, but detectors are subject to high price and require an integrated cooling system^13^. Also, the extended InGaAs and InGaAs/GaAsSb type-II quantum well photodetectors have been intensively studied, but so far cannot detect the full SWIR region^14–17^. Detectors that are of low-cost and fully cover the SWIR region are required.

Indium arsenide (InAs), one of the III-V materials, has a high electron mobility of 30,000 cm^2^/V·s and a direct band gap of 0.417 eV. Due to the narrow band gap, InAs-based detectors can sense SWIR light that is longer than the cut-off wavelength of a conventional InGaAs photodetector. However, although the physical properties of InAs constitute a photodetector that is advantageous, manufacturing the detectors is difficult due to the cost of InAs wafers, which are more expensive than InP wafers.

GaAs substrates are cheaper than InAs and InP substrates; however, the lattice mismatch between GaAs and InAs is 7.2%, which leads to inevitable defects such as misfit dislocations (MDs) and threading dislocations (TDs). The dislocations have a negative impact on the physical properties of InAs due to electron-defect scattering. In order to minimize defects caused by lattice mismatch, it is essential to employ a metamorphic growth technique. For example, these include the low and high growth temperature (LT-HT) In_x_Ga_1−x_As step graded buffer and In_x_Al_1−x_As step graded buffer layer methods^18–20^.

In the LT-HT growth method, the InAs film is grown at LT and then the InAs film is grown at HT. The initial high density InAs nucleation covers the GaAs substrate at LT, and the strain induced by lattice mismatch between the GaAs and InAs relaxes by generating high density dislocations. Subsequently, the LT InAs is reconstructed into a continuous film by heating to HT. Then, the reconstructed LT InAs film acts as a pseudo substrate for the growth of high-quality InAs films^18^. In LT GaAs films grown on an InP substrate, the dislocations generated by stress-induced lattice mismatch are immobilized and the dislocations slide towards a symmetric orientation. As the film thickness increases, the dislocation density decreases by symmetrically oriented dislocation scattering in two dimensions^21^.

In the graded buffer layer method, the stress induced by lattice mismatch can be relaxed through stepwise or linearly constant lattice change, and the dislocation density is decreased due to the hardening of the alloy for an In composition of 0.5 for In_x_Ga_1−x_(Al_1−x_)As^20^.

Recently, Loke *et al*.^22–24^ have reported an InAs photodetector grown on a GaAs substrate using the In_x_Al_1−x_As graded buffer layer and LT-GaAs. The composition x was continuously changed from 0 to 1. The InAs layer was the surface of the graded buffer layer, and was not an insulating layer for the InAs film. This conductive layer prevented the precise electronic characterization of the InAs layer. Moreover, the motion of dislocations, which leads to the degradation of the devices due to carrier-defect scattering, is not directly shown in this report.

In our previous work, a high-quality InSb film was grown on a GaAs substrate using an In_x_Al_1−x_Sb continuous graded buffer layer^25^. Unlike other reports, the growth temperature of the continuous graded buffer layer gradually decreased because the growth temperature of InSb is lower than that of AlSb. Proper control of the growth temperature can minimize slippage and generation of dislocations by increasing yield strength.

In the aforementioned two references, the thickness of the continuous buffer layer was above 1.4 μm. The dislocation density is well known to be inversely proportional to film thickness. However, a thick buffer layer is not desirable for real applications.

In this work, a high-quality InAs film was obtained using an In_x_Al_1−x_As graded buffer layer, with x = 0 to x = 0.87 for the insulating surface of the graded buffer layer. The composition x was gradually changed by varying the growth rate of In and Al, while gradually decreasing the growth temperature. The In_0.87_Al_0.13_As layer closest to the InAs film showed sufficient insulating property to allow precise electronic measurement of the InAs films, and has proper Al composition to be used as sacrificial layer for future integration. In our study, the effect of the In_x_Al_1−x_As graded buffer layer for fabricating high-quality InAs film is better than that of LT-HT buffer layers. To fabricate a SWIR imaging operating at room temperature, the p-i-n InAs was grown on a semi-insulating (SI) GaAs substrate by inserting the In_x_Al_1−x_As graded buffer layer.

## Results and Discussion

**Figure 1:**
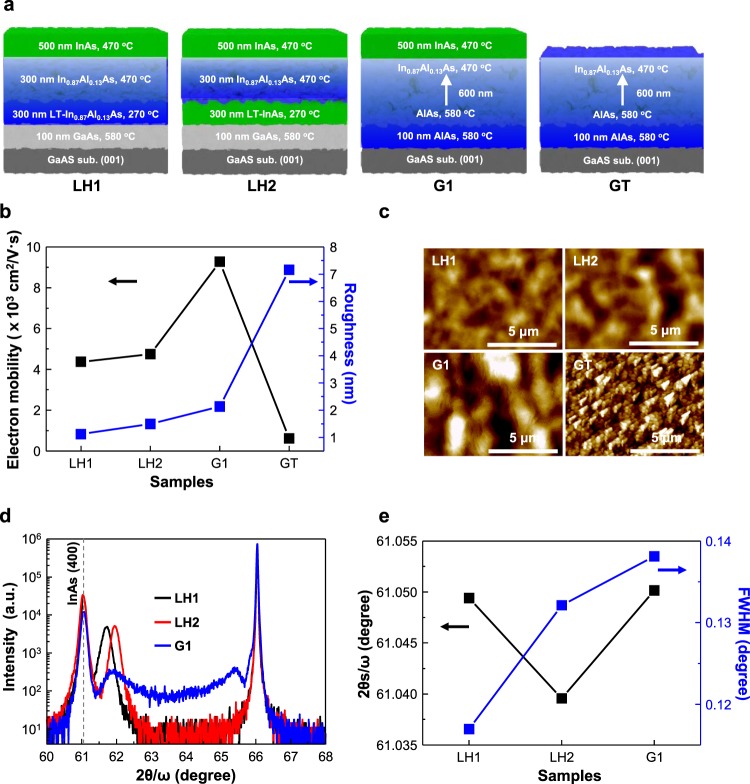
Growth schemes of InAs films on a GaAs substrate and their structural and electrical properties. (**a**) Schemes of sample growth using In_0.87_Al_0.13_As - In_0.87_Al_0.13_As (LH1), InAs - In_0.87_Al_0.13_As (LH2), In_x_Al_1−x_As graded (G1), and In_0.87_Al_0.13_As terminated (GT) buffer layers on GaAs substrates. (**b**) Electron mobility and surface roughness of the samples. (**c**) Surface morphology, obtained atomic force microscopy (AFM), of the samples. The scale bar is 5 μm. (**d**) XRD 2θ/ω spectra of the samples. The dashed line indicates the InAs (400) peak position. (**e**) InAs (400) 2θ/ω peak and its full width at half maximum (FWHM).

Three samples of InAs were grown on SI-GaAs (001) using In_0.87_Al_0.13_As - In_0.87_Al_0.13_As (LH1), InAs - In_0.87_Al_0.13_As (LH2), and In_x_Al_1−x_As graded (G1) buffer layers in a Riber compact 21E solid source molecular beam epitaxy (MBE) system, as shown in Fig. 1a.

In the LH1 and LH2 samples, the GaAs substrate was degassed at 400 °C and then the removal of the native oxide from the substrate was carried out in an As_2_ atmosphere supported by a valved-cracker As cell at the substrate temperature (T_s_) of 620 °C for 10 min. Subsequently, a 100-nm thick GaAs buffer layer having a Ga growth rate of 1.82 Å/s was deposited on a flat surface at 580 °C. An InAs layer of 300 nm thickness was grown in a GaAs buffer layer at the LT of 270 °C and then an In_0.87_Al_0.13_As layer of 300 nm thickness was cultured at an HT of 470 °C in the LH1. Subsequently, an InAs film having a thickness of 500 nm was grown at 470 °C. The growth procedure of LH2 is the same as that of LH1, but with a 300-nm thick In_0.87_Al_0.13_As layer, instead of an InAs film grown at LT. The In growth rate of In_0.87_Al_0.13_As and InAs was 2.62 Å/s, while the Al growth rate of the LT-HT samples was 0.4 Å/s.

During the growth procedure of the G1 samples, the degassed, deoxy, and GaAs buffer layer deposition processes were similar to those mentioned above. Then, a 100 nm thick AlAs layer was deposited with an Al growth rate of 2.7 Å/s at a T_s_ of 580 °C. At that point, an In_x_Al_1−x_As graded buffer layer was grown while gradually changing the growth rate of In from 0 Å/s to 2.62 Å/s, and the Al from 2.7 Å/s to 0.4 Å/s, while the T_s_ was gradually decreased from 530 to 470 °C. Finally, a 500-nm InAs film was grown at a T_s_ of 470 °C.

The structure of the In_0.87_Al_0.13_As terminated (GT) sample is shown in Fig. 1a, and the sample demonstrated an insulating property.

Figure 1b shows the electron mobilities and surface roughness of four samples. The electron mobilities of LH1, LH2, G1, and GT were 4,375, 4,749, 9,275 and 617 cm^2^/V·s, respectively. Apart from the GT sample, the other samples showed the electron mobilities of a 500-nm thick InAs film, and the G1 sample had the highest electron mobility. This implies that the graded buffer layer can overcome the lattice mismatch between GaAs and InAs, and that it is a more efficient approach than the LT-HT growth method for obtaining a high quality InAs film on a GaAs substrate.

The surface roughness of LH1, LH2, G1, and GT was 1.1, 1.5, 2.1, and 7.2 nm, respectively, and obtained images of 10 μm × 10 μm, as shown in Fig. 1c. Although the surface roughness of G1 was higher than that of LH1 and LH2, the electron mobility of G1 was also higher than that of LH1 and LH2. This means that surface scattering does not have a major influence in the degradation of carrier transport, taking into account the electron mobilities and surface roughness.

The degradation of electron mobility ascribes to electron-defect scattering, and several hillocks and pits in LH1 and LH2 are slightly higher than that in G1, as shown in Fig. 1c. The high density of hillocks and pits has a negative impact on electron transport. The surface morphology of GT is the roughest among the samples, and the pyramidal surface morphology implies that the graded buffer layer could grow at low growth temperature. Interestingly, the high surface roughness of the graded buffer layer reduced from 7.2 nm to 2.1 nm after the 500 nm thick InAs grew in the GT. The electron mobility of the GT was 617 cm^2^/V·s, and its resistivity of 18 Ω/cm was also higher than the G1 resistivity of 0.015 Ω/cm (data not shown). Therefore, the electron mobility of the 500-nm thick InAs film was precisely achieved due to the insulating property of the GT layer.

The 2θ/ω spectra of samples are shown in Fig. 1d. The In_0.87_Al_0.13_As 2θ/ω peaks in LH1 and LH2 were at 61.71° and 61.94°, respectively, while the GaAs (004) peak was 66.05°. The 2θ/ω angle difference of In_0.87_Al_0.13_As between LH1 and LH2 is attributed to the In and Al growth rates, which was measured at high temperature. The growth rate varied at low temperature. In particular, the In growth rate rapidly increased due to a reduction in the re-evaporation of In at low temperature.

For G1, the peak of the In_x_Al_1−x_As graded buffer layer was widely obtained in the range of 65.5° to 61.85°. The InAs (004) peak and its full width at half maximum (FWHM) are shown in Fig. 1e. The InAs (004) peaks of LH1, LH2, and G1 were positioned at 61.08°, 61.04°, and 61.05° and the lattice constant from the peaks was 6.064 Å, 6.067 Å, and 6.066 Å, respectively. The lattice constants are slightly larger than those of InAs, at 6.058 Å, and the epitaxial InAs films are almost relaxed. The threading dislocation density of the InAs film in the G1 sample is 9.4 × 10^7^/cm^2^, which was calculated by Ayer’s model in XRD spectra.

The 500 nm thick InAs film in G1 was lattice mismatched to the GaAs by 7.32% out of plane. This means that the lattice of InAs films was stretched out of the plane. The FWHM of G1 was wider than that of LH1 and LH2, while the G1 sample showed the highest electron mobility among these samples. The electron-defect scattering of the G1 sample was minimized due to the relatively low defect density in these samples. Therefore, the stress induced by the lattice mismatch between InAs and GaAs relaxes by the deformation of the atomic structure of InAs in the G1 sample, while the stress relaxes by sliding and the generation of dislocations in the LH1 and LH2 samples.

**Figure 2:**
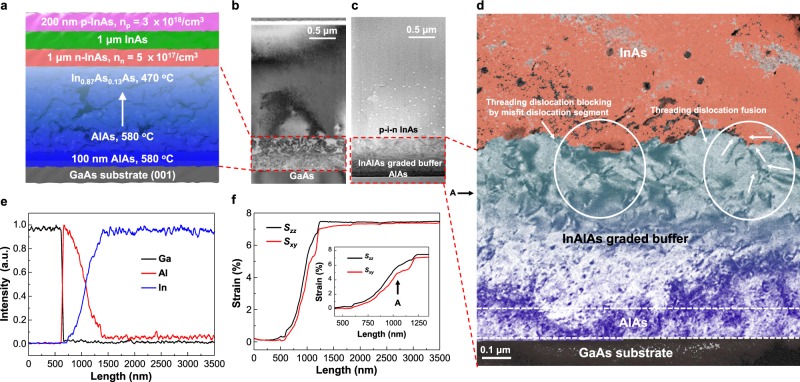
Structural diagram and micro structure analysis of p-i-n inas on a GaAs substrate. (**a**) Scheme of the p-i-n InAs on a GaAs substrate. (**b**) Transmission electron microscopy (TEM) dark-filed image of p-i-n InAs on a GaAs substrate. (**c**) High angle annular dark field (HAADF) image of p-i-n InAs on a GaAs substrate. (**d**) High-resolution TEM image of the InAs/InAlAs graded buffer/AlAs/GaAs substrate. The A point indicates the change in the rate of increase in the in-plane strain, as shown in the strain line profile (**f**). The image shows threading dislocations blocked by misfit dislocation segments, and threading dislocation fusions in white circles. (**e**) Energy-dispersive X-ray spectroscopy (EDS) elemental line profile of p-i-n InAs on a GaAs substrate. (**f**) In-plane and out-of-plane strain line profile of p-i-n InAs on a GaAs substrate. The A point indicates the point in the TEM image (**d**).

The InAs p-i-n photodetector sample grew on an In_x_Al_1−x_As graded buffer layer in the G1 scheme. The p- and n-type InAs film grew with Be and Si at a doping concentration of 3 × 10^18^/cm^3^ and 5 × 10^17^/cm^3^, respectively. The structural scheme and the TEM dark-field image of the p-i-n InAs film are shown in Fig. 2a,b, respectively. The AlAs and In_x_Al_1−x_As layers are not clearly distinguishable, but the thickness of the layers in total is approximately 730 nm. Also, a high density of defects is shown in the layers.

Meanwhile, the thickness of the InAs p-i-n layer was 2370 nm, and some edge dislocations appeared as defects in the TEM image. Therefore, the graded buffer layer suppressed the nucleation and sliding of the dislocations, inducing a lattice mismatch between the InAs film and the GaAs substrate, and the dislocations confines to the graded buffer layer.

The brightness in a HAADF image indicates the number of electrons scattered inelastically by the atoms in a sample, and the heavy atoms appear to be brighter than the light atoms. Figure 2c exhibits a high angle annular dark field (HAADF) image of the InAs p-i-n sample with an AlAs layer of ~110 nm, and distinctly shows the InAlAs layer of ~620 nm in thickness due to the difference in brightness. Furthermore, the brightness increases from the surface of the AlAs layer to that of the In_x_Al_1−x_As graded buffer layer, which implies that the Al and In compositions have gradually changed in the buffer layer. The white circles dissipate In rich droplets during the focused ion beam (FIB) milling process in the InAs p-i-n region. The interface between the In_x_Al_1−x_As graded buffer and the InAs p-i-n layer is rugged and corresponds to the pyramidal shaped surface of the GT AFM image, as shown in Fig. 1c.

The limited In diffusion results in a difference in growth rates along the (001) and (111) faces due to the atomic density in these directions. The low growth temperature limits dislocation motion and increases yield strength^26,27^. Thus, structural deformation is more beneficial than the sliding and nucleation of the dislocations to mitigate stress caused by the lattice mismatch and induced by the low growth temperature. Similarly, the dislocations reflect in the interface between the InAs and InAlAs graded buffer layer.

The TEM image of the InAs/In_x_Al_1−x_As graded buffer/AlAs/GaAs, obtained by two beam conditions, is shown in Fig. 2d, and the black circles in the InAs are the In rich droplets. The misfit and threading dislocations appear in the lower region of the In_x_Al_1−x_As graded buffer layer, and the dislocations generated in the lower region have slipped. In this case, the dislocation density decreases with increasing film thickness. Meanwhile, the dislocation density in the upper region of the In_x_Al_1−x_As graded buffer layer decreases as the InAs layer closes through threading dislocation blocking by misfit dislocation segment and fusion process. In addition, the dislocations in the upper region of the In_x_Al_1−x_As graded buffer layer almost do not slip directly to the InAs layer. In other words, the dislocations are confined in the In_x_Al_1−x_As graded buffer layer. The localization of the dislocations is attributed to the abrupt interface^28,29^. The confinement of dislocations is also shown in Fig. 2b, where point A is explained by Fig. 2f.

The normalized EDS line profile is illustrated in Fig. 2e. The amount of Al and In gradually decreases and increases, respectively, along the growth direction, as expected with an In_x_Al_1−x_As graded buffer layer. The change in the In and Al composition in the graded buffer layer is consistent with the increased brightness in the HADDF image shown in Fig. 2c. As noted, this brightness relates to the atomic mass.

The strain line profile of the InAs p-i-n sample is shown in Fig. 2f and is relatively obtained to the lattice constant of GaAs substrate by the Topspin experiment. The inset of Fig. 2f shows the enlarged strain profile of the In_x_Al_1−x_As layer in the sample. Here, *s*_*xy*_ and *s*_*zz*_ indicate the in-plane (110) and out-of-plane (001) strain, respectively. The *s*_*zz*_ in the AlAs layer increases slightly, while *s*_*xy*_ has almost no change. This means that the stress induced by the lattice mismatch between the GaAs and AlAs is alleviated by the deformation of the AlAs cubic structure. The *s*_*xy*_ and *s*_*zz*_ gradually increase in the In_x_Al_1−x_As graded buffer layer, and the increased rate of strain is conformable, from the AlAs to point A.

The similar increase in the rates of *s*_*xy*_ and *s*_*zz*_ in the lower region of point A attributes to stress relaxation caused by the generation and sliding of dislocations. On the other hand, the *s*_*zz*_ rate of increase is faster than that of *s*_*xy*_ in the upper region of point A. The difference in rates between *s*_*xy*_ and *s*_*zz*_ is attributed to the deformation of the In_x_Al_1−x_As cubic structure. Therefore, the stress in the upper region of point A is dominantly relieved by the deformation of the cubic structure rather than the generation and sliding of dislocations. In other words, for stress relaxation, the generation and sliding of the dislocations is advantageous in the lower part of point A, while the deformation of the cubic structure is advantageous in the upper part of point A.

At the interface between the graded buffer layer and the InAs layer, *s*_*zz*_ and *s*_*xy*_ are 7.45% and 7%, respectively. The *s*_*zz*_ is in good agreement with the XRD result, which is approximately 7.32%. As the thickness of InAs increases, the difference between the *s*_*xy*_ and *s*_*zz*_ strain gradually decreases until the *s*_*zz*_ and *s*_*xy*_ are about 7.45% and 7.35%, respectively, at the surface of the InAs layer. Although the strain of InAs against GaAs is larger than the original strain of 7.25%, the gradual equalization of the strains implies that the atomic structure of InAs has recovered from the deformed cubic to the cubic form.

**Figure 3:**
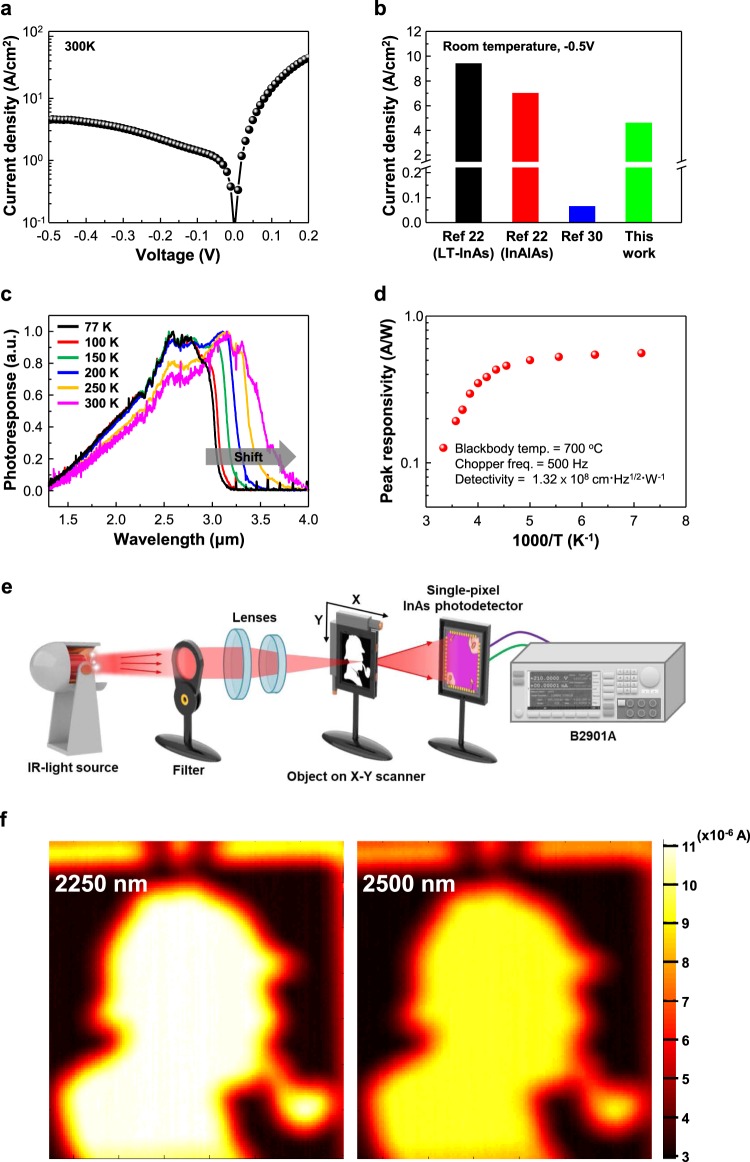
Performance of the InAs photodetector. (**a**) Current density-voltage (*J-V*) curve of the fabricated InAs photodetector, measured at room temperature. (**b**) Dark current density values reported by others, at a bias of – 0.5 V at room temperature. (**c**) Photoresponse of the InAs photodetector at various temperatures. The cut-off wavelength is simultaneously red-shifted by increasing temperature. (**d**) Peak responsivity of the InAs photodetector at various temperatures, measured by a blackbody temperature of 700 °C and chopper frequency of 500 Hz. The detectivity (D^*^) is obtained using Eq. (1). (**e**) Schematic illustration of the single-pixel imaging system in a transmissive configuration used for 2D imaging in the short-wave infrared range. (**f**) 2D photo-detection images at the peak wavelengths of 2.25 μm and 2.5 μm, filtered by band-pass filter.

Figure 3 shows the electrical and opto-electrical properties of the fabricated detector. Figure 3a exhibits the current density versus bias (J-V) curve of the detector measured at room temperature, and shows the rectification characteristics of the p-i-n junction. The dark current density of the InAs photodetector, measured at room temperature under a bias of −0.5 V, is 4.6 A/cm^2^ as shown in Fig. 3b. It is slightly lower than those of other InAs photodetectors (PDs) on a GaAs substrate. The dark current densities of InAs PDs, which were grown using a LT-InAs and an InAlAs graded buffer layer, are 9.4 A/cm^2^ and 7 A/cm^2^, respectively^22^. Furthermore, it is worth noting that these values are approximately two orders of magnitude higher than that of 0.064 A/cm^2^ from a homo-epitaxial InAs photodetector due to the dependence of the dark current density on the dislocation density^30^.

Figure 3c shows the normalized photoresponse of the detector as a temperature variation, and the cut-off wavelength is red-shifted from about 3.1 μm at 77 K to 4 μm at 300 K. The changed cut-off wavelengths relates to the band gap of InAs at various temperatures. The peak responsivity is shown in Fig. 3d and its value varies from 0.6 A/W at 80 K to 0.126 A/W at 300 K. The detectivity (D^*^) of the photo-detector is given by

1 $${D}^{\ast }={R}_{p}\,{(\frac{{R}_{0}A}{4kT})}^{1/2}$$

where *k*, *T*, and *A* are Boltzmann’s constant, temperature, and the window of the device, respectively.

The D^*^ of the InAs photodetector at room temperature is estimated by Eq. (1) to be 1.65 × 10^8^ cm · Hz^1/2^ · W^−1^ at 3.3 μm.

In order to verify the imaging capability of the InAs photodetector, 2D image scanning was performed by means of a bi-axial mechanical scanner. Figure 3e depicts a schematic diagram of a single-pixel imaging system in a transmissive configuration used for 2D imaging in the short wave infrared (SWIR) range. Since the InAs photodetector was used as a single-pixel sensor, a bi-axial mechanical scanner was used to obtain 2D images. Also, two linear stages (Physik Instrument, Q-545) were used to scan the x and y axes, respectively. The 2D photo-detection mapping image was obtained using a filtered IR-light source with a band-pass filter, and an openwork thin Sherlock Holmes bookmark was used as a scanning object. The position of the scanned object was in the plane where the image of the pinhole spot was formed by the use of lenses. Therefore, the output images can be obtained by scanning the spot through the scanned object. The scanned object was attached to the x-y planar moving stage, and the object was scanned and captured by the single-pixel InAs photodetector. A source measurement unit (Keysight Technologies, B2901A) was used to measure photocurrents. Each pixel value corresponds to the current measurement obtained at the corresponding scanner position.

The 2D mapping images of the photo-detection are shown in Fig. 3f. A band-pass filter with an FWHM of 0.5 ± 0.1 μm filters the light source. The 2D mapping image with a 2250 nm filter is brighter than that of a 2500 nm filter, but a stronger photoresponse is attributed to the spectral dependence of the light-source power. Despite the differences in photoresponse, the Sherlock Holmes image is clearly displayed, which means that the InAs p-i-n photodetector, grown using the In_x_Al_1−x_As graded buffer layer, can be used as an image sensor for the MIR region.

## Conclusion

To obtain a high quality InAs film, we investigated an InAs film grown on a GaAs substrate using a LT-HT and graded buffer layer. The InAs film with the graded buffer layer exhibited the highest electron mobility among the films and its value is 9,275 cm^2^/V·s. The InAs p-i-n photodetector on a GaAs substrate grew using an In_x_Al_1−x_As graded buffer layer (x = 0 → 0.87). The cut-off wavelength of the detector is approximately 4 μm and the responsivity is 0.126 A/W at room temperature.

In future work, we will focus on the research of the integration of InAs photodetectors with wafer bonding and epitaxial lift-off method, because the InAlAs/AlAs graded buffer was designed for selective etching^31^. Additionally, the InAs photodetector will be optimized.

## Methods

Electrical properties such as resistivity, carrier mobility, and carrier concentration were measured by an Ecopia HMS-3000 Hall Measurement System with an input current of 0.1 mA at room temperature.

The InAs surface and morphology were measured using a Park systems XE-100 with an NSC-36 tip in non-contact mode.

The lattice spacing on the out-of-plane samples was measured using a Rigaku ATX-G XRD.

The microstructure was studied using a TECNAI F20 G2 SuperTwin TEM (FEI, Hillsboro, OR) with the sample mounted in a double-tilt holder (Gatan, Pleasanton, CA). TEM images were obtained by the Fischhione Model 3000 ADF detector and UltraScan 1000 (2k × 2k) CCD camera (Gatan Inc.). Energy dispersive spectroscopy (EDS) and TopSpin analysis, which are strain analysis tools, were performed using a Talos F200X TEM with Bruker Super EDS system and NanoMEGAS DigiSTAR precession electron diffraction (PED) unit with Appfive software in TENCAI F20 G2 SuperTwin TEM, respectively.

The current density of the 500 × 500 μm detector was measured at 300 K by a Keithley 4200 integrated in a probe station. The photocurrent spectra were obtained using a customized system with a Bruker VERTEX 80 v Fourier transform infrared spectrometer, a global MIR source, a Janis cryo-chamber, and a Keithley 428 low-noise current amplifier. The peak responsivity was measured using a fully-integrated smart home system with a 700 °C blackbody source, vacuum chamber, and SR830 lock-in amplifier for a 77 K low temperature experiment.

For 2D imaging, the optical wavelength range from an IR-source (SLS202L, Thorlab) was filtered by a band-pass filter (FB2250-500 or FB2500-500, Thorlab), and then the optical path was passed to the single InAs p-i-n photodetector or masked by a Sherlock Holmes bookmark supported by an XY automated stage. The experiment was carried out at room temperature.

## Supplementary information

InAs on GaAs Photodetectors Using Thin InAlAs Graded Buffers and Their Application to Exceeding Short-Wave Infrared Imaging at 300K
