# Supplementary material for: InAs on GaAs Photodetectors Using Thin InAlAs Graded Buffers and Their Application to Exceeding Short-Wave Infrared Imaging at 300 K
Source: Sci Rep. 2019 Sep 6;9:12875. doi: 10.1038/s41598-019-49300-z (PMC6731284; doi:10.1038/s41598-019-49300-z)
Supplement: Supplementary file 1 — InAs on GaAs Photodetectors Using Thin InAlAs Graded Buffers and Their Application to Exceeding Short-Wave Infrared Imaging at 300K [file 41598_2019_49300_MOESM1_ESM.pdf]

Supplementary Information

InAs on GaAs Photodetectors Using Thin InAlAs Graded Buffers and Their Application to Exceeding Short-Wave Infrared Imaging at 300K

Soo Seok Kang[1,2], Dae-Myeong Geum[1], Kisung Kwak[1], Ji-Hoon Kang[1], Cheol-Hwee Shim[3], HyeYoung Hyun[3], Sang Hyeon Kim[1], Won Jun Choi[1], Suk-Ho Choi[2], Min-Chul Park[1,], and Jin Dong Song[1,*]*

1. Center for Opto-Electronic materials and devices, Korea Institute of Science and Technology, Seoul 136-791, Republic Korea

2. Department of Applied Physics and Institute of Natural Sciences, Kyung Hee University, Yongin 17104, Republic Korea

3. Advanced Analysis Center, Korea Institute of Science and Technology, Seoul 136-791, Republic Korea

Corresponding author: minchul@kist.re.kr or jdsong@kist.re.kr

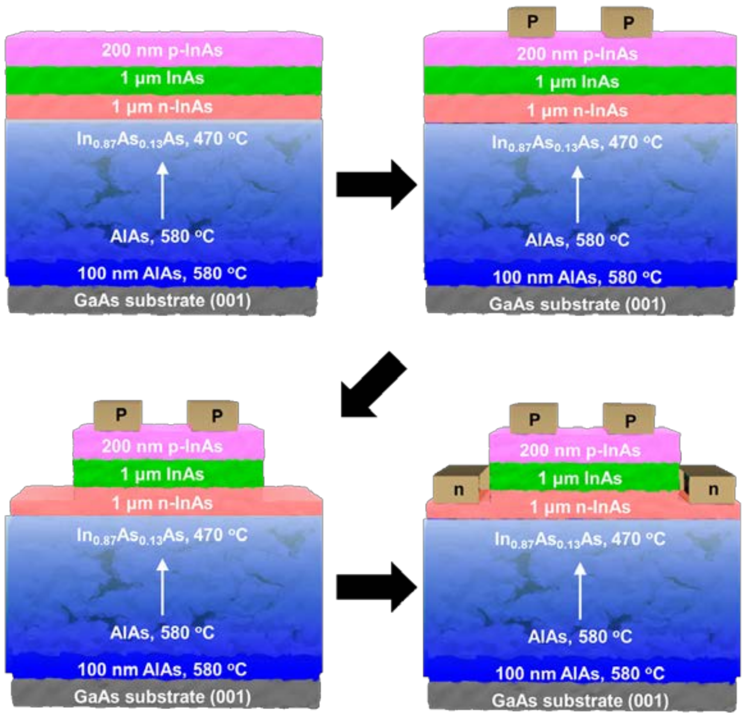

Figure S1. Fabrication process of a p-i-n InAs photodetector

The p-i-n InAs photodetector was fabricated using the following process as shown in Supplemental Figure S1. The p-i-n InAs epitaxial layer was cleaned by acetone, methanol, and deionized (DI) water for 10, 5, and 5 minutes, respectively. The surface native oxide of the epitaxial layer was removed by HCl:DI (1:5) solution treatment for 1 minute after photo-lithography for the mesa structure is carried out. The Pt/Ti/Pt/Au for the top electrode were deposited at 20/30/20/300 nm thicknesses by electron beam evaporator, and then the p-type and intrinsic InAs layer was etched by phosphoric acid (H_3PO_4) and hydrofluoric acid (HF) solutions until the n-InAs layer was exposed. The Ni/Au/Ge/Ni/Au for the bottom electrode contact were deposited at 20/100/30/30/300 nm thicknesses.

Finally, the fabricated p-i-n InAs photodetector with a 500 × 500 μm^2 window was investigated for an infra-red detecting application.
